# Using technology for improving population health: comparing classroom vs. online training for peer community health advisors in African American churches

**DOI:** 10.1186/1748-5908-10-S1-A60

**Published:** 2015-08-20

**Authors:** Cheryl L Holt, Sherie L Zara Santos, Erin Kelly Tagai, Mary Ann Scheirer, , Roxanne Carter, Janice V Bowie, Muhiuddin Haider, Jimmy Slade, Min Qi Wang, Tony Whitehead

**Affiliations:** 1 Department of Behavioral and Community Health, University of Maryland, College Park, MD 20742, USA; 2 Scheirer Consulting, Princeton, NJ 08540, USA; 3 Community Ministry of Prince George's County, Upper Marlboro, MD 20773, USA; 4 Johns Hopkins Bloomberg School of Public Health, Baltimore, MD 21205, USA; 5 Institute for Applied Environmental Health, University of Maryland, College Park, MD 20742, USA; 6 Community Ministry of Prince George's County, Upper Marlboro, MD 20773, USA; 7 Department of Behavioral and Community Health, University of Maryland, College Park, MD 20742, USA; 8 University of Maryland, Department of Anthropology, University of Maryland, College Park, MD 20742, USA

## Background

Technology is increasingly used in health promotion interventions. Project HEAL (Health Through Early Awareness and Learning) compared two methods of training lay community health advisors (CHAs): 1) the traditional/classroom approach vs. 2) a new online training system.

## Methods

Fourteen African American churches located throughout Prince George's County, MD were randomized to receive the traditional/classroom (N = 8 churches) or the online (N = 6 churches) training approach. The CHAs received Project HEAL workshop intervention materials and led a 3-part workshop series in his/her church encouraging breast, prostate, and colorectal cancer early detection. Study participants completed surveys at workshops 1 and 3 and another at 12 months post-baseline, which were used to evaluate the intervention impact on Health Belief Model-based outcomes and self-reported cancer screening.

## Results/Findings

CHAs recruited 385 African Americans age 40 -75 (122 men and 261 women) with an average age of 54.75 (SD = 9.16), and 229 (60%) completed the 3-month follow-up survey. The intervention resulted in significant overall pre-post increases in colorectal cancer knowledge (p < 0.05), and prostate cancer knowledge (p < 0.001), yet knowledge specific to the prostate cancer "controversy" decreased (p < 0.05). In addition, participants expressed significantly greater satisfaction (e.g., interest; personal relevance; importance; trust) with the workshops from the online approach than in the classroom approach (p < 0.01). Finally, the men taught by online-trained CHAs had marginally greater increases in prostate cancer knowledge as compared to those taught by the classroom-trained CHAs (p < 0.07), and this effect became significant at 12 months (p < 0.05). Additional analyses examine change in baseline to 12-month follow in study outcomes.

## Discussion

Use of an online approach is a novel way to train peer community health advisors and has implications for wider scalability and reach. We discuss lessons learned and implications for dissemination/implementation research in this context.

This research is funded by the National Cancer Institute (#R01CA147313). None of the authors have any commercial interests.

